# Cs1, a *Clonorchis sinensis*-derived serodiagnostic antigen containing tandem repeats and a signal peptide

**DOI:** 10.1371/journal.pntd.0006683

**Published:** 2018-08-02

**Authors:** Na Cheng, Xue-Nian Xu, Yan Zhou, Yu-Ting Dong, Yi-Fang Bao, Bin Xu, Wei Hu, Zheng Feng

**Affiliations:** 1 National Institute of Parasitic Diseases, Chinese Center for Disease Control and Prevention, Shanghai, People’s Republic of China; 2 WHO Collaborating Centre for Tropical Diseases, Key Laboratory of Parasite and Vector Biology, National Health and Family Planning Commission, Shanghai, People’s Republic of China; 3 State Key Laboratory of Genetic Engineering, Ministry of Education Key Laboratory of Contemporary Anthropology, Collaborative Innovation Center for Genetics and Development, School of Life Sciences, Fudan University, Shanghai, People’s Republic of China; Baylor College of Medicine, UNITED STATES

## Abstract

**Background:**

Clonorchiasis, caused by the liver fluke *Clonorchis sinensis*, remains a serious public health issue in Asia, especially in China, and its relationship with cholangiocarcinoma has highlighted the importance of *C*. *sinensis* infection. Proteins containing tandem repeats (TRs) are found in a variety of parasites and, as targets of B-cell responses, are valuable for the serodiagnosis of parasite infections. Here, we identified a novel *C*. *sinensis*-specific antigen, Cs1, containing TRs, and investigated its diagnostic value, other immunological properties, and tissue distribution.

**Methodology/Principal findings:**

A partial Cs1 cDNA sequence was cloned by screening an adult *C*. *sinensis* cDNA expression library. The full-length Cs1 cDNA was obtained by 5′ rapid amplification of cDNA ends. The deduced Cs1 protein consists of a signal peptide and five TRs of 21 amino acids. The recombinant Cs1 (rCs1) was constructed and purified. rCs1 showed higher sensitivity (94.3%) and specificity (94.4%) than the *C*. *sinensis* excretory–secretory products (ESPs) according to ELISA of 114 serum samples. Native Cs1 was identified in *C*. *sinensis* ESPs and crude antigens of adult *C*. *sinensis* by western blotting using an anti-rCs1 monoclonal antibody. ELISA of recombinant peptides of different Cs1 regions demonstrated that the TR region was immunodominant in Cs1. Immunohistochemistry and confocal microscopy revealed that Cs1 is located in a granule-like structure surrounding the acetabulum of *C*. *sinensis* adults that has not previously been described.

**Conclusions/Significance:**

We identified a novel *C*. *sinensis*-specific TR protein, Cs1, which is an antigen of high serological significance, compared with *C*. *sinensis* ESPs. The deduced features of Cs1 show a unique structure containing TRs and a signal peptide and the TR region is immunodominant in Cs1. This provides a basis for targeted screens of other antigens. The novel structure in which Cs1 is located also deserves further investigation.

## Author summary

Clonorchiasis is a neglected tropical disease. The major factor that prevents the effective management of clonorchiasis is a lack of effective diagnostic tools. Proteins containing tandem repeats (TRs), which have been found in a variety of parasites, are known targets of B-cell responses and can be useful for the serodiagnosis of parasite infections. Here we identified a novel *C*. *sinensis*-specific cDNA, which we named Cs1. This cDNA encodes a protein that has a unique structure, containing TRs and a signal peptide. A recombinant Cs1 protein (rCs1) was expressed and purified. rCs1 showed a high sensitivity and specificity in enzyme-linked immunosorbent assays, and lower cross-reactivity with *Paragonimus westermani* compared with *C*. *sinensis* excretory–secretory products. Our results also indicated that the TR region was immunodominant in the Cs1 protein. Immunohistochemistry and confocal microscopy revealed that Cs1 was located in a granule-like structure surrounding the acetabulum of adult worms that has not been previously described in *C*. *sinensis*. These results show that Cs1 is a promising antigen for serodiagnosis of clonorchiasis and its features provide a basis for future targeted screens of entire proteomes based on the likelihood of seroreactivity.

## Introduction

Clonorchiasis is a food-borne parasitic disease caused by infection with the liver fluke *Clonorchis sinensis*, which is mainly endemic in China, South Korea, northern Vietnam, and parts of Russia. It was conservatively estimated that in 2004 around 15 million people were infected, of whom an estimated of 13 million are in China [1–4].

*C*. *sinensis* infection causes liver and biliary diseases. An increased risk of developing cholangiocarcinoma, a malignant tumor that arises from the bile ducts, is the most severe clinical manifestation. *C*. *sinensis* was classified as a Group 1 biological carcinogenic agent (carcinogen) by the International Agency for Research on Cancer in 2009 [5]. Clonorchiasis is an urgent public health problem in most endemic areas [6, 7], and is included in the control programs of neglected tropical diseases by the World Health Organization.

For many years the diagnosis of clonorchiasis has been primarily based on fecal examination, using the Kato–Katz method and the formalin–ether technique, which are labor-intensive and time-consuming, especially for mass screening in the field. A variety of immunological and serological approaches, including enzyme-linked immunosorbent assay (ELISA) and indirect fluorescence antibody tests, have been used as supplementary tests. The current ELISA is a reliable diagnostic test for clonorchiasis that uses crude extracts or excretory–secretory products (ESPs) of adult worms [8–10]. *C*. *sinensis* ESPs are thought to be superior to crude extracts, showing a sensitivity of 93.1% [8]. However, it is difficult to produce sufficient amounts of ESPs. Thus, the serodiagnostic applicability of several recombinant proteins (7-kDa protein, CsLPAP, CsEF-1α, 28-kDa cystein protease, and 26-kDa and 28-kDa glutathione S-transferases) from *C*. *sinensis* worms has recently been evaluated [11–16]. These recombinant proteins show a range of sensitivities and specificities for the serodiagnosis of clonorchiasis, but are not sufficient to replace crude extracts or ESPs. Thus, further research is needed to identify more effective serological antigens.

To determine whether alternative *C*. *sinensis*-specific antigens could be used, we serologically screened a *C*. *sinensis* expression library using pooled sera from clonorchiasis patients. This identified a novel *C*. *sinensis*-specific cDNA that we named Cs1. This cDNA encodes a protein with a signal peptide and tandem repeats (TRs). We also characterized the biochemical and immunological properties and immunolocalization of Cs1 protein.

## Methods

### Ethics statement

The animal experiment was reviewed and approved by the Animal Welfare & Ethics Committee of the National Institute of Parasitic Diseases, Chinese Center for Disease Control (NIPD, China CDC) (Permit No: IPD-2009-14) (S1 Fig) and followed the National Guidelines for Experimental Animal Welfare (MOST of People’s Republic of China, 2006). Ethical clearance for the collection and analysis of human samples was obtained from the Ethical Review Committee of the NIPD, China CDC (Permit No: 20120826) (S2 Fig). Written informed consent for participation in this study was obtained from all clonorchiasis patients and healthy controls (all subjects were adults). Archived human sera used in this study were obtained from the serum samples library of the NIPD, China CDC.

### Parasites and human sera

*C*. *sinensis* metacercariae were obtained from naturally infected *Pseudorasbora parva* captured from endemic areas in Guangxi Province, China, as previously described [17], and were orally administered to New Zealand white rabbits. Adult worms were collected from rabbit bile ducts 6 weeks post-infection. ESPs were prepared as follows. Adult worms were cultured in Tyrode’s solution with penicillin (100 U/ml) and streptomycin (100 μg/ml). The culture supernatant was collected every 24 h, centrifuged at 1000 × g for 10 min at 4°C, aliquoted and stored at −80°C. Crude antigens of *C*. *sinensis* adult worms were prepared as previously described [8].

A total of 114 serum samples were collected from patients with clonorchiasis (egg-positive as detected by the modified Kato–Katz technique) (n = 35), schistosomiasis japonica (n = 15), paragonimiasis westermani (n = 15), or cysticercosis (n = 13) from areas of China where each parasite is endemic, and from healthy individuals without parasitic infections (n = 36) from Guangxi Province, China (an *C*. *sinensis* endemic area). All sera were stored at –80°C until used.

### Serological screening of a *C*. *sinensis* expression library

An adult *C*. *sinensis* cDNA expression library was constructed as previously described [17]. The cDNA library, 1.1 × 10^6^ pfu, was mixed with *Escherichia coli* XL1-Blue and cultured on a LB-agar plate. The plate was overlaid with a nitrocellulose (NC) membrane (Amersham, UK), which was treated previously with 10 mM isopropyl-D-thiogalactoside (IPTG), and then incubated at 37°C for 4 h. The membrane was further incubated for 3 h with pooled sera of five clonorchiasis patients diluted 1:100 at room temperature (RT), followed by incubation with the goat anti-human IgG alkaline phosphatase-conjugated secondary antibody (Sigma, USA) diluted 1:2,000. A color signal was developed with the addition of 5-bromo-4-chloro-3-indolyl phosphate and nitro blue tetrazolium (Sigma, USA) solution. Positive clones were purified through secondary and tertiary screenings using the same sera. These clones were then excised *in vivo* to single-stranded phagemids by employing ExAssist helper phage (Stratagene, USA), and converted to double-stranded plasmids according to the manufacturer’s instructions.

The positive clones were sequenced and analyzed, and sequence homology was searched in NCBI using BLAST (http://blast.ncbi.nlm.nih.gov/Blast.cgi).

### Identification of the full-length Cs1 cDNA sequence

A partial cDNA sequence of Cs1 was obtained through screening the adult *C*. *sinensis* cDNA expression library. To determine the full-length Cs1 cDNA sequence, the 5′ RACE System for Rapid Amplification of cDNA Ends (Invitrogen, USA) was used to amplify Cs1 transcripts from mRNAs of adult worms, using GSP1: 5′-TGCGCACCATCCGCATCG-3′ for cDNA synthesis, GSP2: 5′-GATGTGCTCGAGCCTGAAG-3′ and AAP primer (see instruction manual) for PCR amplification. PCR products were analyzed by electrophoresis on a 1% agarose gel and then cloned into the pGEM-T Easy Vector (Promega, UK) and sequenced.

### Bioinformatic analysis

Cs1 and other serologically screened genes (reported in our previous studies [18–21]) from the adult *C*. *sinensis* cDNA expression library were analyzed with Tandem Repeats Finder (http://tandem.bu.edu/trf/trf.html) as previously described [22] to determine whether they are TR genes. However, TR domains in the deduced proteins were manually identified because some of the TR sequences in these genes did not match with coding regions. The biochemical characteristics of their deduced amino acid sequences were analyzed to determine: (i) the molecular mass, isoelectric point, and amino acid composition using ProtParam (http://web.expasy.org/protparam/), (ii) the presence of a signal sequence using the SignalP 4.1 Server (http://www.cbs.dtu.dk/services/SignalP/), and (iii) the presence of a transmembrane domain using the TMHMM Server v. 2.0 (http://www.cbs.dtu.dk/services/TMHMM/).

### Cloning, expression, and purification of recombinant Cs1 (rCs1), rCs1C+TR, rCs1N, and rCs1TR

The coding region of Cs1 (the N-terminal signal peptide sequence was omitted) was amplified by PCR using the positive phagemid corresponding to Cs1 (pBlueScript-SK-Cs clone 5) as the template, and forward primer 5′-CCGACATGTCTGAGGACATTTTAG-3′ and reverse primer 5′-CCCAAGCTTTGATATGATTCTTCGTAGAATT-3′. These primers have *Pci*I and *Hind*III sites, respectively, at their 5′-ends (underlined). The specific PCR product was purified and digested with *Pci*I and *Hind*III (all the restriction endonucleases used in this study were from New England Biolabs, USA), and recombined into the pET28a expression vector (Promega, USA) digested with *Nco*I and *Hind*III. The construct was confirmed by DNA sequencing and then transformed into BL21 (DE3)-competent cells (TIANGEN, China). The expression of recombinant Cs1 (rCs1) was induced by IPTG (1 mmol/L). rCs1 was purified under non-denaturating conditions from culture supernatant using an Ni-NTA affinity column (QIAGEN, USA) according to the manufacturer’s instructions. The efficiencies of rCs1 expression and purification were analyzed by 12% sodium dodecyl-sulfate-polyacrylamide gel electrophoresis (SDS-PAGE).

The TR region and two flanking regions (the N-terminus not including the signal peptide, and the C-terminus plus the TR region because the C-terminus is too short to be expressed) of Cs1 were amplified by PCR, using PET28a-rCs1 as the template, and specific primer sets as follows: TR region, 5′-CATGCCATGGAATGTGACCCTGAATCAG-3′ and 5′-CCGCTCGAGAATCTCACCTTGCGGTTT-3′, N-terminus (not including the signal peptide), 5′-CCGACATGTCTGAGGACATTTTAG-3′ and 5′-TTTTCCTTTTGCGGCCGCGTCACATTCTCCTTTCACTG-3′, C-terminus plus TR region, 5′-CATGCCATGGAATGTGACCCTGAATCAG-3′ and 5′-CCGCTCGAGTGATATGATTCTTCGTAGAATT-3′ (restriction sites are underlined). The amplified PCR products (Cs1TR and Cs1C+TR) were digested with *Nco*I and *Xho*I, respectively, and then inserted into pET28a digested with the same restriction enzymes. Cs1N was digested with *Pci*I and *Not*I, and then inserted into pET28a digested with *Nco*I and *Not*I. Recombinant peptides (named rCs1C+TR, rCs1N, and rCs1TR, respectively) were expressed and purified as for rCs1.

### ELISA

The diagnostic value of rCs1 was evaluated by ELISA (rCs1-ELISA) and compared with that of *C*. *sinensis* ESPs (ESP-ELISA). Briefly, 96-well plates were coated with ESPs (2.5 μg/well) or rCs1 (0.5 μg/well) in 0.05 M bicarbonate buffer, pH 9.6 (100 μl/well) overnight at 4°C. Human sera were reacted with proteins at a 1:100 dilution for 1 h at 37°C. After washing with phosphate-buffered saline/0.05% Tween 20 (PBST, pH 7.4), goat anti-human IgG conjugated with horseradish peroxidase (Sigma, USA) was incubated at 1:10,000 for 1 h at 37°C. All experiments were carried out in duplicate. The cutoff value was set as the mean value plus two standard deviations of the absorbance of negative control samples.

Sensitivity is represented by Se = TP/(TP+FN), where TP (true positive) is the number of sera from individuals infected with *C*. *sinensis* above the cutoff value, and FN (false negative) is the number of sera from infected individuals below the cutoff for the conserved peptide. Specificity is represented by Sp = TN/(TN+FP), where TN (true negative) is the number of sera from un-infected individuals below the cutoff, and FP (false positive) is the number of sera from these samples with reactivity.

The serological reactivity of rCs1C+TR, rCs1N, and rCs1TR were valued using the same method.

### Preparation of monoclonal antibodies against rCs1

Monoclonal antibodies were produced in BALB/c mice using rCs1 as an antigen as previously described [16, 23, 24]. Briefly, 8-week-old female BALB/c mice were each immunized with 50 μg rCs1 in Freund’s complete adjuvant and boosted twice with the same amount of the mixture of incomplete adjuvant every 2 weeks later. The final boost was given intravenously without adjuvant. Spleen cells were then isolated from the immunized mice 3 days later and fused to SP2/0 plasmacytoma cells. Hybridoma culture supernatants were screened by ELISA. ELISA-positive hybridoma cell lines were cloned at least 3 times by limiting dilution prior to large-scale production. The cloned hybridoma cells, 5×10^6^ in number, were inoculated intraperitoneally into the BALB/c mouse and IgG antibody was purified from ascites by protein G agarose (Thermo scientific, USA). The ascites of mice injected with SP2/0 plasmacytoma cells was prepared as a negative control (SP2/0).

### Western blotting

ESPs or crude antigens of *C*. *sinensis* adult worms were subjected to 12% SDS-PAGE, then electrotransferred onto a polyvinylidene fluoride (PVDF) membrane (Millipore, USA) at 100 V for 1 h in a Trans-Blot transfer cell (Bio-Rad, USA). The membrane was incubated with an anti-rCs1 monoclonal antibody (mAb; Cs1-2-6-3) at 1:200 dilution, and subsequently in horseradish peroxidase-conjugated goat anti-mouse IgG (1:5,000; Sigma, USA). Finally, the color was developed with diaminobenzidine substrate solution.

### Immunohistochemical assay

To determine the localization of Cs1 in *C*. *sinensis* adult worms, they were fixed in 4% paraformaldehyde, embedded in paraffin, and sliced at 6–7 μm thicknesses. Paraffin sections were deparaffinized, rehydrated, and then restoration heat-treated. The sections were incubated with anti-rCs1 mAb (Cs1-2-6-3) diluted 1:20 in PBS for 2 h at 37°C, with SP2/0 used as a negative control. The sections were washed three times with PBS and then incubated with fluorescein isothiocyanate-conjugated anti-mouse IgG (Sigma, USA) for 1 h at 37°C in the dark. After washing, sections were observed under a fluorescence microscope (Olympus BX51).

### Three-dimensional (3D) localization of Cs1 using confocal microscopy

To further determine the 3D localization of Cs1, *C*. *sinensis* adult worms were leaf-shaped in 10% neutral formalin overnight at 4°C, and then washed twice with 5% sucrose/PBS, twice with PBS, three times with 5% TritonX-100/PBS, and three times with 1% TritonX-100/PBS. The worms were then blocked with 3% bovine serum albumin/PBS for 1 h at RT, and incubated with anti-rCs1 mAb (Cs1-2-6-3) diluted 1:50 in PBS for 1.5 h at RT; SP2/0 was used as a negative control. The worms were washed three times with 1% TritonX-100/PBS and then incubated with Alexa Flour 488 (AF488)-conjugated anti-mouse IgG (Sigma, USA) for 1 h at RT in the dark. After washing three times with 1% TritonX-100/PBS, worms were counterstained with propidium iodide (PI, 10 μg/ml, with 10 μg/ml RNase A) for 0.5 h at 37°C in the dark. After washing three times with 1% TritonX-100/PBS, worms were observed under a laser confocal microscope (Nikon C2). Images were captured and analyzed using reconstruction software (NIS-Elements).

### Statistical analysis

Statistical analyses were performed using SPSS for Windows, version 19.0 (SPSS Inc., Chicago, IL). The chi-square test was used for the analysis of significance. The statistical significance was defined as *P* < 0.05.

## Results

### Serological screening of the *C*. *sinensis* expression library

A total of 44 positive clones were obtained through screening the adult *C*. *sinensis* cDNA expression library using pooled sera from five clonorchiasis patients. These clones were sequenced and divided into six categories according to their sequence similarities (S1 Table). Two encoded previously well-characterized antigens, GRCSP (glycine-rich antigen 1) and cysteine proteinase, three categories were reported in our previous studies [18–21], and the remaining category, Cs1, is reported here.

### Sequence analysis of Cs1, a novel *C*. *sinensis*-specific antigen containing a signal peptide and TRs

**Fig 1:**
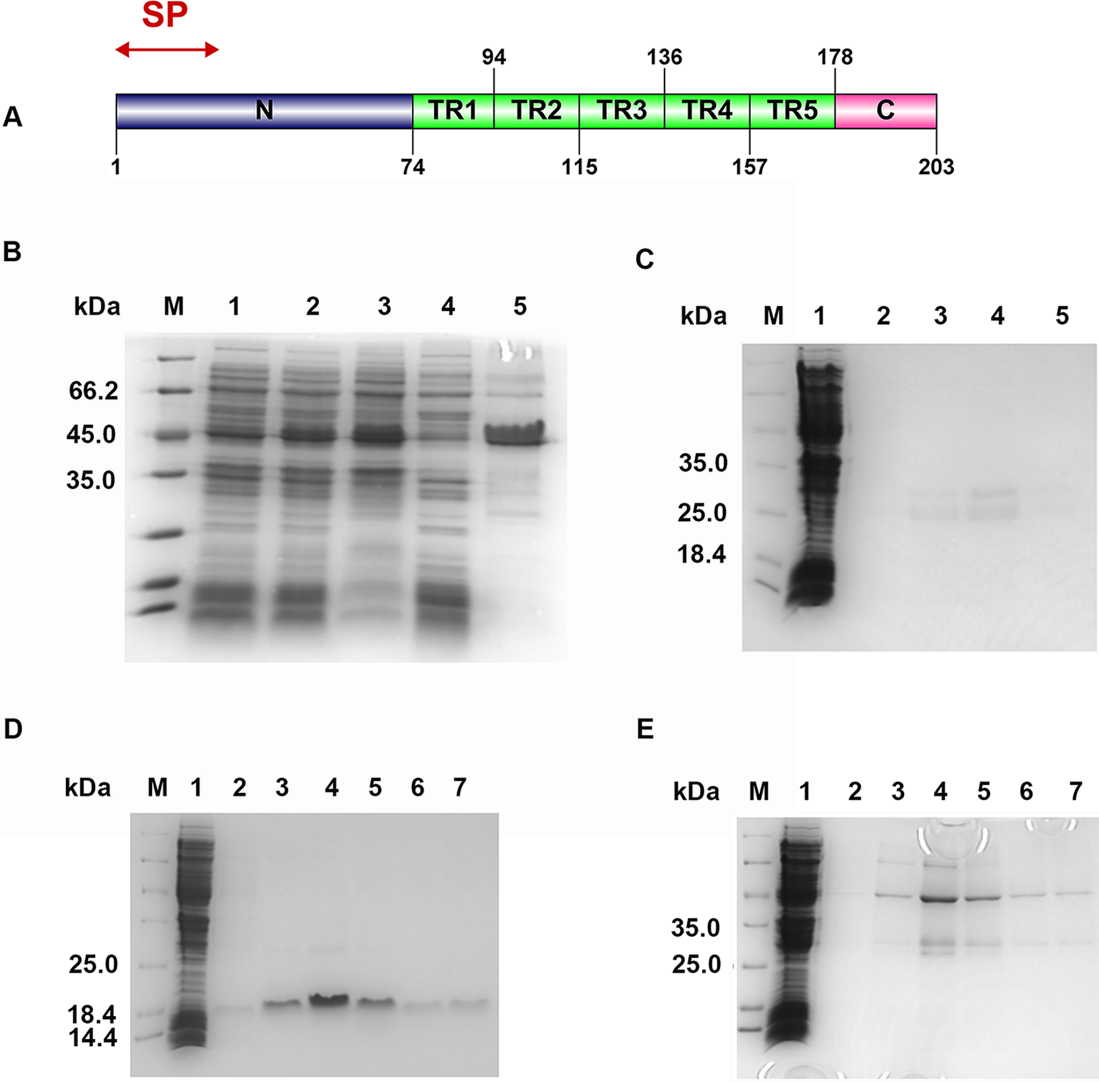
The predicted molecular properties of Cs1 protein and expression of different fragments of Cs1. **(A)** The predicted molecular properties of Cs1 protein. SP: signal peptide; N: N-terminus; TR: tandem repeat; C: C- terminus. **(B)** Expression and purification of rCs1 by 12% SDS-PAGE. *Lane 1*, BL21(DE3) cells containing the recombinant pET28α-Cs1 homolog without IPTG induction; *lane 2*, BL21(DE3) cells containing the recombinant pET28α-Cs1 homolog with IPTG induction; *lane 3*, supernatant of lysed BL21(DE3) cells containing the recombinant pET28α-Cs1 homolog with IPTG induction; *lane 4*, pellet of lysed BL21(DE3) cells containing the recombinant pET28α-Cs1 homolog with IPTG induction; *lane 5*, the recombinant protein purified by affinity purification. **(C)** Expression and purification of rCs1TR. *Lanes 1–5*, different fractions of purified rCs1TR protein. **(D)** Expression and purification of rCs1N. *Lanes 1–7*, different fractions of purified rCs1N. **(E)** Expression and purification of rCs1C+TR. *Lanes 1–7*, different fractions of purified rCs1C+TR. *Lane M* in (B)-(E) shows molecular mass standards.

The full-length Cs1 cDNA sequence was obtained by 5′-RACE and consists of 733 nucleotides, with a coding sequence from 23–619 that encodes a putative protein of 198 amino acids. No orthologue was found in the *Opisthorchis viverrini* genome archived in the GenBank database. BLAST results showed that Cs1 has a very low level of similarity (≤11%) with other genes, indicating that it is novel and *C*. *sinensis*-specific. The deduced Cs1 protein is composed of a signal peptide (MGMKPQLVYFIFIQLVTAECLA) and five complete TRs of 21 amino acids (DPESDGAVADDAPPSQVKPQG) (Fig 1A).

The Cs1 cDNA sequence was submitted to GenBank (accession number: HM236312.1).

### Serological reactivity of rCs1 compared with ESPs

The PCR product of the Cs1 coding region produced a 550 bp band by agarose gel electrophoresis (Panel A in S3 Fig). rCs1 was expressed mainly in the supernatant of *E*. *coli*, with an apparent molecular weight of 45 kDa (Fig 1B).

**Fig 2:**
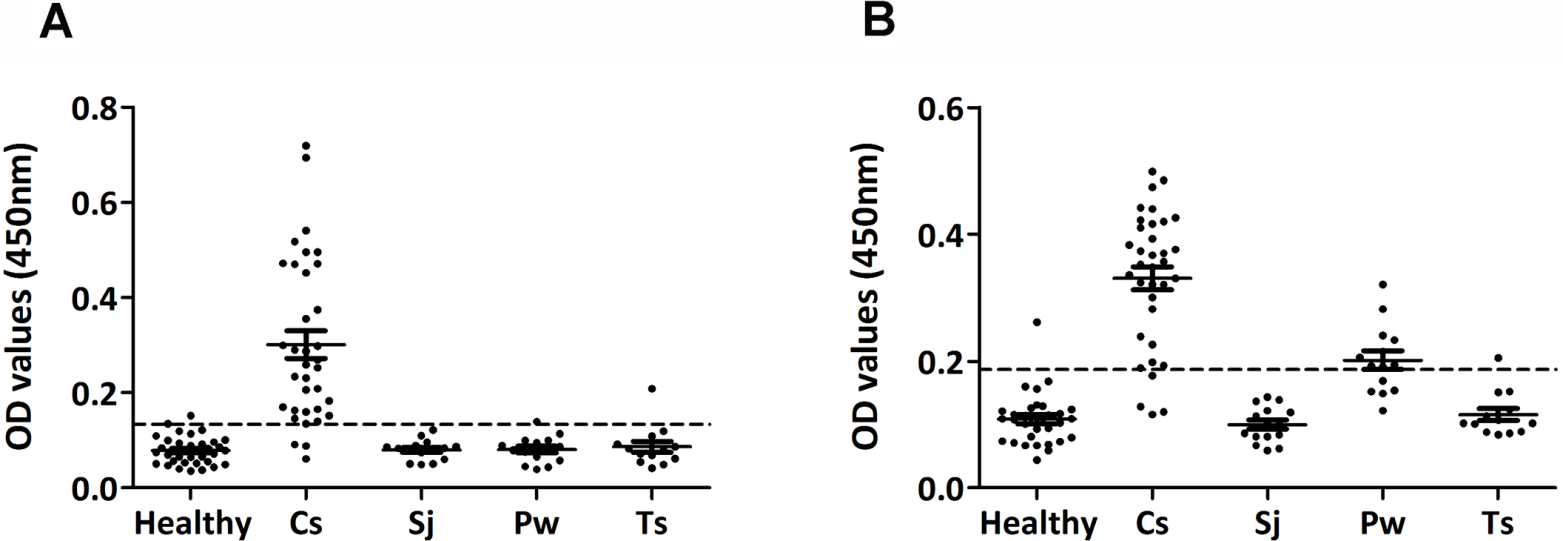
Comparison of the sensitivity and specificity of rCs1-ELISA and ESP-ELISA. **(A) rCs1-ELISA. (B) ESP-ELISA.** The two methods were used to assay a total of 114 serum samples, including 35 samples infected with clonorchiasis (Cs) and 36 uninfected samples (Healthy), 15 samples infected with schistosomiasis japonica (Sj), 15 samples infected with paragonimiasis westermani (Pw) and 13 samples infected with cysticercosis (Ts). OD = optical density.

ELISA was used to compare the diagnostic value of rCs1 (rCs1-ELISA) with that of *C*. *sinensis* ESPs (ESP-ELISA). A total of 114 serum samples were assayed, including sera from patients with clonorchiasis (n = 35), schistosomiasis japonica (n = 15), paragonimiasis westermani (n = 15), and cysticercosis (n = 13), and from healthy individuals (n = 36). rCs1-ELISA showed a lower cut-off compared with ESP-ELISA. Moreover, rCs1-ELISA showed 6.7% cross-reactivity with *Paragonimus westermani*, while ESP-ELISA showed 60.0% cross-reactivity with *Paragonimus westermani* (*P* < 0.01). Overall, rCs1-ELISA showed 94.3% (33/35) (95% CI: 90.0% ~ 98.6%) sensitivity and 94.9% (75/79) (95% CI: 90.9% ~ 98.9%) specificity, compared with 88.6% (31/35) (95% CI: 82.8% ~ 94.4%) and 86.1% (68/79) (95% CI: 79.7% ~ 92.5%), respectively, for ESP-ELISA (Fig 2).

### Identification of Cs1 as a component of both *C*. *sinensis* ESPs and crude antigens by western blotting

**Fig 3:**
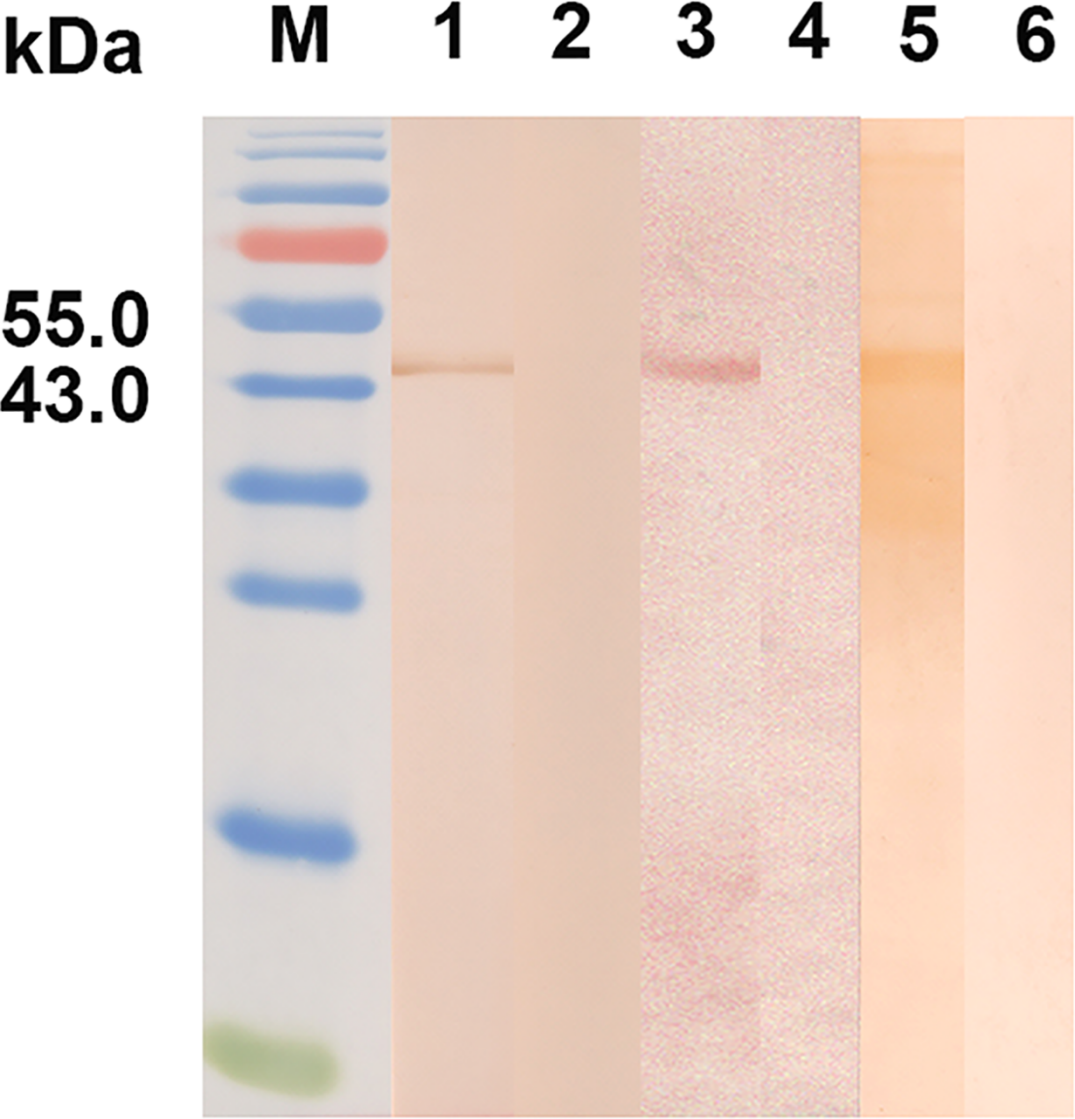
Western blotting of recombinant and native Cs1. Blots were probed with anti-rCs1 monoclonal antibody (Lanes 1, 3, and 5) or S/P20 (Lanes 2, 4, and 6). Lanes 1 and 2, rCs1; Lanes 3 and 4, *C*. *sinensis* ESPs; Lanes 5 and 6, crude antigens of adult *C*. *sinensis*. M, protein molecular weight marker.

The Cs1 protein was predicted to have a signal peptide and was predicted to be secreted by the parasite. Western blotting showed that rCs1 was recognized by the anti-rCs1 monoclonal antibody (mAb; Cs1-2-6-3). Cs1-2-6-3 recognized the native Cs1 protein of 45 kDa in ESPs and crude antigens of *C*. *sinensis* adult worms (Fig 3).

These results indicate that Cs1 is a component of both the ESPs and crude antigens of *C*. *sinensis*.

### Immunological dominance of Cs1 TR

To examine whether the TR region is immunodominant in the Cs1 protein, the complete TR region, the N-terminus (without signal peptide), and the C-terminus plus TR region of Cs1 were amplified (Panel B in S3 Fig) and then expressed as recombinant peptides (rCs1TR, rCs1N and rCs1C+TR), with apparent molecular weights of 28 kDa, 19 kDa, 30 kDa, respectively (Fig 1C–1E). These proteins were then evaluated by ELISA using the pooled sera from patients with clonorchiasis (n = 10), and pooled sera from healthy individuals (n = 10) as a negative control.

rCs1TR and rCs1C+TR both showed positive reactions (P/N > 2.1, P: patient, N: negative control), while rCs1N showed a negative reaction (P/N < 2.1). These results indicate that the TR region is immunodominant in the Cs1 protein.

### Abundance of strongly acidic amino acids in the Cs1 TR

The PI value of the Cs1 protein is 4.07. The TR amino acid sequence of Cs1 is DPESDGAVADDAPPSQVKPQG. These 21 amino acids include five negatively-charged residues (four D, one E) and one positively-charged residue (one K). These results indicate that the TR region in Cs1 protein has an abundance of strongly acidic amino acids.

### Immunolocalization of Cs1

To determine the tissue distribution of Cs1, immunohistochemistry and confocal microscopy were performed on *C*. *sinensis* adult worms using an anti-rCs1 mAb (Cs1-2-6-3).

**Fig 4:**
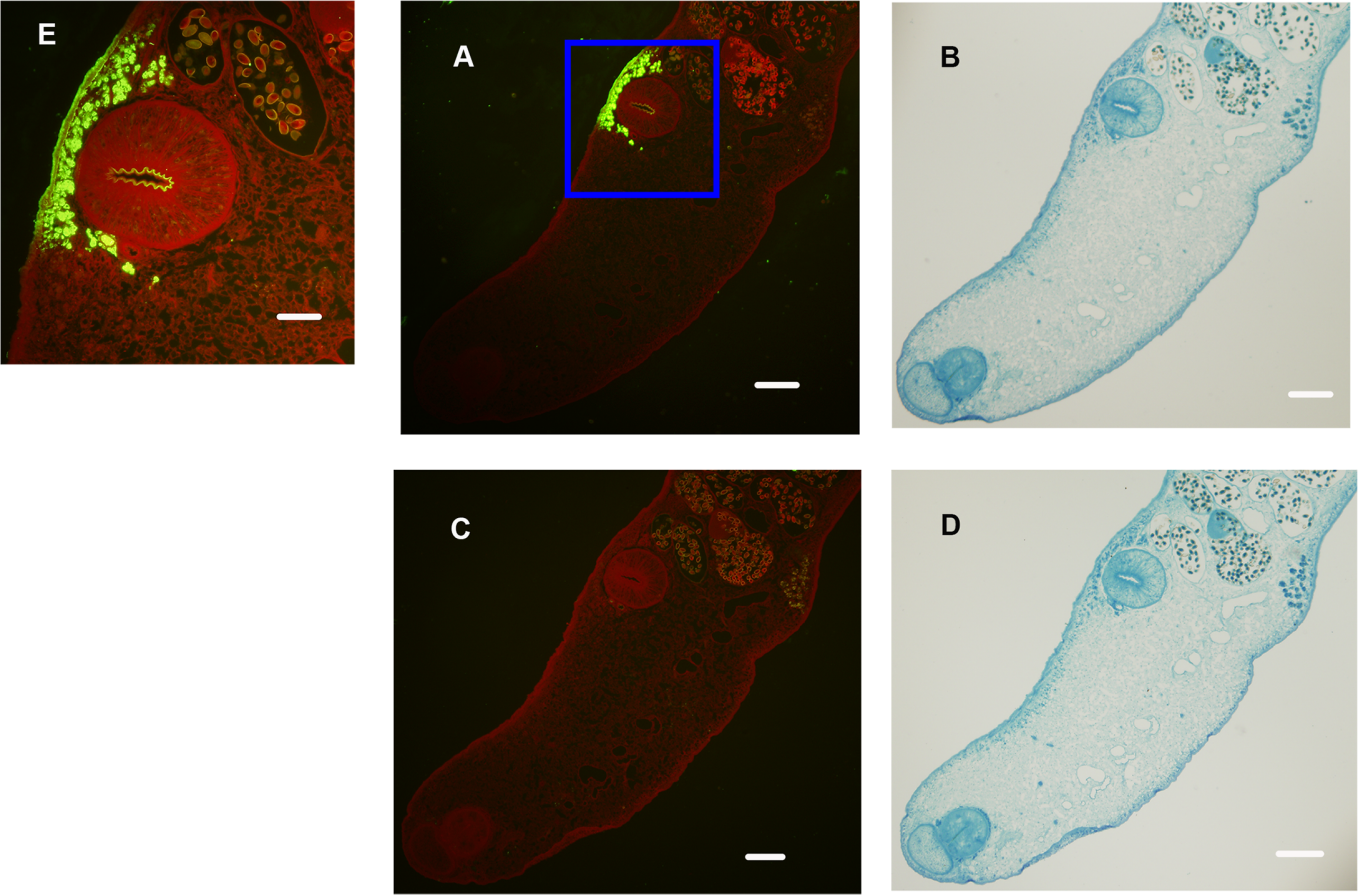
Immunolocalization of Cs1 in adult *C*. *sinensis*. Fluorescence microscopy images (left) and corresponding optical images (right) of adult worms are shown. Cs1 localization is represented in (A) and (B) and the negative controls are shown in (C) and (D). (E) is 2.5 times the magnification of the boxed area in (A). Scale bar equals 500 μm in (A)-(D) and 250 μm in (E).

Sections of *C*. *sinensis* adult worms were probed with Cs1-2-6-3 (Fig 4A and 4B) or SP2/0 (Fig 4C and 4D).

A strong positive reaction was observed in the cells in a granulate, gland-like structure surrounding the acetabulum of *C*. *sinensis* adult worms, and a moderately positive reaction was seen in the outer tegument of the acetabulum (Fig 4A and 4E).

**Fig 5:**
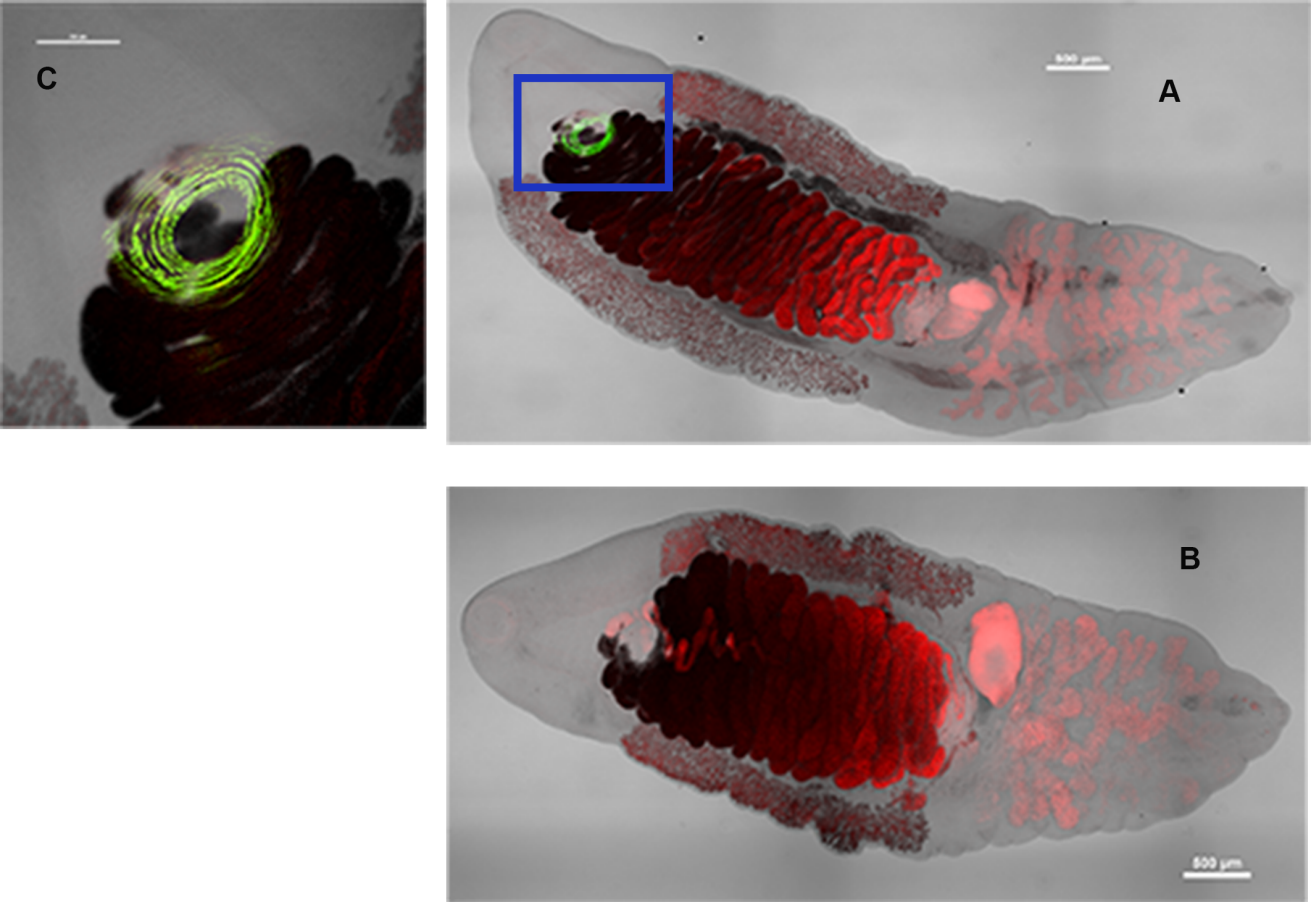
Localization of Cs1 (green) in adult *C*. *sinensis* by confocal microscopy. *C*. *sinensis* adults were incubated with (A) anti-rCs1 mAb (Cs1-2-6-3) or (B) negative control (S/P20). (C) is 2.5 times the magnification of the boxed area of (A). Scale bar equals 500 μm in (A) and (B), and 250 μm in (C).

Confocal microscopy results further confirmed that Cs1 is located in the cells surrounding the acetabulum of adult *C*. *sinensis* worms, and showed it to have a dish-like structure (Fig 5).

## Discussion

In the present study, we identified a novel *C*. *sinensis*-specific TR protein for the serodiagnosis of clonorchiasis. The deduced Cs1 sequence (198 amino acids) is composed of a signal peptide and five TRs of 21 amino acids. No orthologue was found in the genome of *O*. *viverrini*, which has a very close relationship to *C*. *sinensis*, in the archived GenBank database. BLAST results also showed that Cs1 has low sequence similarity (≤ 11%) with other genes, indicating that Cs1 is novel and *C*. *sinensis*-specific. This result is consistent with previous findings, which demonstrated that many TR proteins are genus- or species-specific [25].

Proteins containing TRs, defined here as proteins consisting of two or more copies of a pattern of amino acids, have been found in a variety of protozoan parasites, including *Plasmodium* [26, 27], *Leishmania* [22, 28–30], *Trypanosoma* [31, 32], and *Fasciola hepatica* [33]. These TR proteins often serve as targets of B cell responses. With respect to *C*. *sinensis*, some currently validated antigens are also TR proteins, including GRCSP (glycine-rich antigen) [34], CsPRA (proline-rich antigen) [35], and pBCs31 [36]. Cs1 is a TR protein containing five complete copies of “DPESDGAVADDAPPSQVKPQG”. Its serodiagnostic value was evaluated by indirect ELISA with a total of 114 human serum samples, and compared with that of *C*. *sinensis* ESPs. Cs1 showed high sensitivity and specificity, which was similar to those of *C*. *sinensis* ESPs. However, *C*. *sinensis* ESPs showed 60% cross reactivity with *P*. *westermani*, whereas the cross reactivity of Cs1 was 6.7% (*P* < 0.01). rCs1-ELISA also showed a lower cut-off compared with ESP-ELISA. ESPs are well-known as serodiagnostic antigens, but our present results indicate that rCs1 may be a potential substitute of ESPs for the diagnosis of clonorchiasis.

To further evaluate the potential of Cs1 for serodiagnosis of opisthorchiasis, ELISA was used to compare Cs1 (rCs1-ELISA) with *C*. *sinensis* ESPs (ESP-ELISA) using serum samples from patients with opisthorchiasis viverrini (n = 5). rCs1-ELISA showed no cross-reactivity with *O*. *viverrini*, while ESP-ELISA showed 60.0% cross-reactivity with *O*. *viverrini* (*P* < 0.01). This result indicates that Cs1 is very specific for *C*. *sinensis* and could be used to discriminate between clonorchiasis and opisthorchiasis. The fact that no orthologue of Cs1 was found in the genome of *O*. *viverrini* also validates this conclusion.

Our studies also demonstrated that the TR region was immunodominant in the Cs1 protein. This is consistent with previous reports demonstrating that proteins containing TR domains are often B cell antigens, and that antibody responses toward TR domains are dominant in humans infected with certain parasites. The overall immunogenicity of proteins harboring TRs is high, as is the antigenicity of epitopes contained within these repetitive units [37–40]. These units may provide additional antigen-presenting sites and antibody-combining sites, which increase the overall immunogenicity and the antigenicity of TR proteins.

The Cs1 TR region has an abundance of strongly acidic amino acids, including five negatively-charged and one positively-charged residue. These results are consistent with previous findings showing that B cell epitopes are rich in negatively-charged residues Asp (D) and Glu (E), positively-charged residues Lys (K), Arg (R), and His (H), non-polar aliphatic residues Gly (G) and Pro (P), polar non-charged residues Asn (N) and Gln (Q), and the aromatic residue Tyr (Y) [37, 38, 41].

The expected size of Cs1 itself is around 20 kDa, but the recombinant or the native protein in crude preparations is much bigger than that. It is not unusual for the molecular weight of a protein on an SDS PAGE gel to be different from its predicted size. In many cases, this molecular weight difference is attributed to chemical modifications of the protein. However, the contribution of chemical modifications was ruled out because the recombinant protein was expressed in a prokaryotic expression system. Another possible reason is that the protein existed as a multimer. However, in theory, multimers should be dissociated by SDS-PAGE. The most probable reason is that Cs1 is an acidic protein with a predicted isoelectric point of 4.07, which resulted in a much larger molecular weight on an SDS-PAGE gel compared with its predicted size. The three truncated fragments of Cs1 (i.e. rCs1N, rCs1TR, and rCs1C+TR) also exhibited much larger molecular weights compared with those predicted (6.6 kDa, 11.9 kDa, and 14.2 kDa, respectively). They are also all acidic proteins with predicted isoelectric points of 4.61, 3.64, and 4.07, respectively. These results are consistent with previous findings showing that proteins rich in negatively-charged residues Asp (D) and Glu (E) have drastically retarded gel mobility[42–45].

All six antigen categories identified from the adult *C*. *sinensis* cDNA library were analyzed for common features. Surprisingly, all six contained signal peptides, and all except cysteine proteinase contained TR domains (S1 Table). This indicates that proteins with TRs and signal peptides may be potent antigens in *C*. *sinensis*, and potentially in other parasites. These features provide a basis for future targeted screens of entire proteomes based on the likelihood of seroreactivity. With respect to Cs1, the features of TR content, SignalP > 0.7, and PI < 5 were also validated by studies in other organisms [39, 40].

In conclusion, we have cloned and expressed Cs1, and assessed its serological properties. We also demonstrated that the repetitive domains of TR proteins are strong serological antigens, and may be useful for the serodiagnosis of *C*. *sinensis*. The unknown granule-like structure surrounding the acetabulum of *C*. *sinensis* adult worms deserves further study to help understand the biological functions of Cs1.

## Supporting information

S1 Fig Ethical clearance for the animal experiment. (PDF)

S2 Fig Ethical clearance for the collection and analysis of human samples. (PDF)

S3 Fig PCR analysis of Cs1 and different fragments of Cs1. (A) Cs1 (without signal peptide). (B) The complete TR region, N-terminus (without signal peptide), and C-terminus plus TR region of Cs1. M, molecular weight marker.
(TIF)

S1 Table Characteristics of genes identified by screening the cDNA library. (DOCX)

## References

[1] Qian MB, Utzinger J, Keiser J, Zhou XN. Clonorchiasis. Lancet (London, England). 2016;387(10020):800–10. 10.1016/s0140-6736(15)60313-0 26299184

[2] Qian MB, Chen YD, Liang S, Yang GJ, Zhou XN. The global epidemiology of clonorchiasis and its relation with cholangiocarcinoma. Infectious diseases of poverty. 2012;1(1):4 10.1186/2049-9957-1-4 23849183 PMC3710150

[3] Lun ZR, Gasser RB, Lai DH, Li AX, Zhu XQ, Yu XB, et al Clonorchiasis: a key foodborne zoonosis in China. Lancet Infect Dis. 2005;5(1):31–41. 10.1016/S1473-3099(04)01252-6 15620559

[4] Diseases COotNSotIHP. A national survey on current status of the important parasitic diseases in human population. Zhongguo Ji Sheng Chong Xue Yu Ji Sheng Chong Bing Za Zhi. 2005;23(5 Suppl):332–40. 16562464

[5] Bouvard V, Baan R, Straif K, Grosse Y, Secretan B, El Ghissassi F, et al A review of human carcinogens—Part B: biological agents. The Lancet Oncology. 2009;10(4):321–2. 19350698 10.1016/s1470-2045(09)70096-8

[6] Keiser J, Utzinger J. Emerging foodborne trematodiasis. Emerg Infect Dis. 2005;11(10):1507–14. 10.3201/eid1110.050614 16318688 PMC3366753

[7] Sripa B, Kaewkes S, Sithithaworn P, Mairiang E, Laha T, Smout M, et al Liver fluke induces cholangiocarcinoma. PLos Med. 2007;4(7):e201 10.1371/journal.pmed.0040201 17622191 PMC1913093

[8] Choi MH, Park IC, Li S, Hong ST. Excretory-secretory antigen is better than crude antigen for the serodiagnosis of clonorchiasis by ELISA. Korean J Parasitol. 2003;41(1):35–9. 10.3347/kjp.2003.41.1.35 12666728 PMC2717480

[9] Lee MK, Hong SJ, Kim HR. Seroprevalence of tissue invading parasitic infections diagnosed by ELISA in Korea. Journal of Korean medical science. 2010;25(9):1272–6. 10.3346/jkms.2010.25.9.1272 20808668 PMC2923801

[10] Kim YJ, Lee SM, Choi GE, Hwang SH, Kim HH, Lee EY, et al Performance of an enzyme-linked immunosorbent assay for detection of Clonorchis sinensis infestation in high- and low-risk groups. Journal of clinical microbiology. 2010;48(7):2365–7. 10.1128/JCM.02506-09 20421441 PMC2897507

[11] Zhao QP, Moon SU, Lee HW, Na BK, Cho SY, Kong Y, et al Evaluation of Clonorchis sinensis recombinant 7-kilodalton antigen for serodiagnosis of clonorchiasis. Clinical and diagnostic laboratory immunology. 2004;11(4):814–7. 10.1128/CDLI.11.4.814-817.2004 15242967 PMC440603

[12] Hu F, Yu X, Ma C, Zhou H, Zhou Z, Li Y, et al Clonorchis sinensis: expression, characterization, immunolocalization and serological reactivity of one excretory/secretory antigen-LPAP homologue. Experimental parasitology. 2007;117(2):157–64. 10.1016/j.exppara.2007.04.003 17507009

[13] Kim TY, Cho PY, Na JW, Hong SJ. Molecular cloning and phylogenetic analysis of Clonorchis sinensis elongation factor-1alpha. Parasitology research. 2007;101(6):1557–62. 10.1007/s00436-007-0676-7 17674047

[14] Nagano I, Pei F, Wu Z, Wu J, Cui H, Boonmars T, et al Molecular expression of a cysteine proteinase of Clonorchis sinensis and its application to an enzyme-linked immunosorbent assay for immunodiagnosis of clonorchiasis. Clinical and diagnostic laboratory immunology. 2004;11(2):411–6. 10.1128/CDLI.11.2.411-416.2004 15013996 PMC371220

[15] Hong SJ, Kim TY, Kang SY, Yu JR, Song KY, Cho SY. Clonorchis sinensis: immunolocalization of 26 kDa glutathione S-transferase in adult worms. Experimental parasitology. 2002;102(3–4):191–3. 12856316 10.1016/s0014-4894(03)00056-0

[16] Kang SY, Ahn IY, Park CY, Chung YB, Hong ST, Kong Y, et al Clonorchis sinensis: molecular cloning and characterization of 28-kDa glutathione S-transferase. Experimental parasitology. 2001;97(4):186–95. 10.1006/expr.2001.4606 11384162

[17] Hong SJ, Seong KY, Sohn WM, Song KY. Molecular cloning and immunological characterization of phosphoglycerate kinase from Clonorchis sinensis. Mol Biochem Parasitol. 2000;108(2):207–16. 10838223 10.1016/s0166-6851(00)00220-6

[18] Liu Q, Xu XN, Zhou Y, Cheng N, Dong YT, Zheng HJ, et al [Molecular cloning and characterization of a novel Clonorchis sinensis antigenic protein containing tandem repeat sequences]. Zhongguo Ji Sheng Chong Xue Yu Ji Sheng Chong Bing Za Zhi. 2013;31(4):245–50. 24812871

[19] Xu XN, Zhou Y, Dong YT, Tan YG, Bao YF, Xu B, et al [Cloning, expression of PPMP antigen genes of Clonorchis sinensis and immunogenic identification of the recombinant proteins]. Zhongguo Ji Sheng Chong Xue Yu Ji Sheng Chong Bing Za Zhi. 2010;28(6):401–5. 21500524

[20] Zhou Y, Xu XN, Yao KL, Zhang HM, Cheng N, Bao YF, et al [Evaluation of Clonorchis sinensis PPMP I antigen Cs2 recombinant protein for immunodiagnosis of clonorchiasis]. Zhongguo Ji Sheng Chong Xue Yu Ji Sheng Chong Bing Za Zhi. 2011;29(3):172–6. 21970103

[21] CNXXZHZYTYBYZLXBJHLXF Z. Immunological characterization and localization of Cs4 recombinant protein of Clonorchis sinensis, one of the glycine rich antigen 2a gene family member. Guo Ji Yi Xue Ji Sheng Chong Bing Za Zhi. 2011;38(3):135–9. 10.3760/cma.j.issn.1673-4122.2011.03.002

[22] Goto Y, Coler RN, Guderian J, Mohamath R, Reed SG. Cloning, characterization, and serodiagnostic evaluation of Leishmania infantum tandem repeat proteins. Infection and immunity. 2006;74(7):3939–45. 10.1128/IAI.00101-06 16790767 PMC1489730

[23] Johnson DR. Murine monoclonal antibody development. Methods in molecular biology (Clifton, NJ). 1995;51:123–37. 10.1385/0-89603-275-2:123 7581691

[24] Akerstrom B, Brodin T, Reis K, Bjorck L. Protein G: a powerful tool for binding and detection of monoclonal and polyclonal antibodies. Journal of immunology (Baltimore, Md: 1950). 1985;135(4):2589–92. 4031496

[25] Goto Y, Duthie MS, Kawazu S, Inoue N, Carter D. Biased cellular locations of tandem repeat antigens in African trypanosomes. Biochemical and biophysical research communications. 2011;405(3):434–8. 10.1016/j.bbrc.2011.01.048 21241659 PMC3042534

[26] Stahl HD, Crewther PE, Anders RF, Brown GV, Coppel RL, Bianco AE, et al Interspersed blocks of repetitive and charged amino acids in a dominant immunogen of Plasmodium falciparum. Proceedings of the National Academy of Sciences of the United States of America. 1985;82(2):543–7. 3881769 10.1073/pnas.82.2.543 PMC397076

[27] Muralidharan V, Goldberg DE. Asparagine repeats in Plasmodium falciparum proteins: good for nothing? PLoS Pathog. 2013;9(8):e1003488 10.1371/journal.ppat.1003488 23990777 PMC3749963

[28] Goto Y, Coler RN, Reed SG. Bioinformatic identification of tandem repeat antigens of the Leishmania donovani complex. Infection and immunity. 2007;75(2):846–51. 10.1128/IAI.01205-06 17088350 PMC1828517

[29] Burns JM Jr., Shreffler WG, Benson DR, Ghalib HW, Badaro R, Reed SG. Molecular characterization of a kinesin-related antigen of Leishmania chagasi that detects specific antibody in African and American visceral leishmaniasis. Proceedings of the National Academy of Sciences of the United States of America. 1993;90(2):775–9. 8421715 10.1073/pnas.90.2.775 PMC45748

[30] Goto Y, Carter D, Guderian J, Inoue N, Kawazu S, Reed SG. Upregulated expression of B-cell antigen family tandem repeat proteins by Leishmania amastigotes. Infection and immunity. 2010;78(5):2138–45. 10.1128/IAI.01102-09 20160013 PMC2863543

[31] Buscaglia CA, Campetella O, Leguizamon MS, Frasch AC. The repetitive domain of Trypanosoma cruzi trans-sialidase enhances the immune response against the catalytic domain. J Infect Dis. 1998;177(2):431–6. 9466532 10.1086/514199

[32] Goto Y, Carter D, Reed SG. Immunological dominance of Trypanosoma cruzi tandem repeat proteins. Infection and immunity. 2008;76(9):3967–74. 10.1128/IAI.00604-08 18625739 PMC2519453

[33] Trudgett A, McNair AT, Hoey EM, Keegan PS, Dalton JP, Rima BK, et al The major tegumental antigen of Fasciola hepatica contains repeated elements. Parasitology. 2000;121 (Pt 2):185–91. 11085238 10.1017/s0031182099006253

[34] Yang HJ, Park SJ, Im KI, Yong TS. Identification of a Clonorchis sinensis gene encoding a vitellaria antigenic protein containing repetitive sequences. Mol Biochem Parasitol. 2000;111(1):213–6. 11087931 10.1016/s0166-6851(00)00298-x

[35] Kim TY, Kang SY, Ahn IY, Cho SY, Hong SJ. Molecular cloning and characterization of an antigenic protein with a repeating region from Clonorchis sinensis. Korean J Parasitol. 2001;39(1):57–66. 10.3347/kjp.2001.39.1.57 11301591 PMC2721066

[36] Yong TS, Yang HJ, Park SJ, Kim YK, Lee DH, Lee SM. Immunodiagnosis of clonorchiasis using a recombinant antigen. Korean J Parasitol. 1998;36(3):183–90. 10.3347/kjp.1998.36.3.183 9755589 PMC2732929

[37] Kringelum JV, Nielsen M, Padkjaer SB, Lund O. Structural analysis of B-cell epitopes in antibody:protein complexes. Molecular immunology. 2013;53(1–2):24–34. 10.1016/j.molimm.2012.06.001 22784991 PMC3461403

[38] Zhao L, Li J. Mining for the antibody-antigen interacting associations that predict the B cell epitopes. BMC structural biology. 2010;10 Suppl 1:S6 10.1186/1472-6807-10-s1-s6 20487513 PMC2873829

[39] Liang L, Felgner PL. Predicting antigenicity of proteins in a bacterial proteome; a protein microarray and naive Bayes classification approach. Chemistry & biodiversity. 2012;9(5):977–90. 10.1002/cbdv.201100360 22589097 PMC3593142

[40] Carmona SJ, Sartor PA, Leguizamon MS, Campetella OE, Aguero F. Diagnostic peptide discovery: prioritization of pathogen diagnostic markers using multiple features. PloS one. 2012;7(12):e50748 10.1371/journal.pone.0050748 23272069 PMC3522711

[41] Zheng W, Ruan J, Hu G, Wang K, Hanlon M, Gao J. Analysis of Conformational B-Cell Epitopes in the Antibody-Antigen Complex Using the Depth Function and the Convex Hull. PloS one. 2015;10(8):e0134835 10.1371/journal.pone.0134835 26244562 PMC4526569

[42] Poetsch A, Molday LL, Molday RS. The cGMP-gated channel and related glutamic acid-rich proteins interact with peripherin-2 at the rim region of rod photoreceptor disc membranes. The Journal of biological chemistry. 2001;276(51):48009–16. 10.1074/jbc.M108941200 11641407

[43] Guan Y, Zhu Q, Huang D, Zhao S, Jan Lo L, Peng J. An equation to estimate the difference between theoretically predicted and SDS PAGE-displayed molecular weights for an acidic peptide. Scientific reports. 2015;5:13370 10.1038/srep13370 26311515 PMC4550835

[44] Korschen HG, Beyermann M, Muller F, Heck M, Vantler M, Koch KW, et al Interaction of glutamic-acid-rich proteins with the cGMP signalling pathway in rod photoreceptors. Nature. 1999;400(6746):761–6. 10.1038/23468 10466724

[45] Hofmann SL, Goldstein JL, Orth K, Moomaw CR, Slaughter CA, Brown MS. Molecular cloning of a histidine-rich Ca2+-binding protein of sarcoplasmic reticulum that contains highly conserved repeated elements. The Journal of biological chemistry. 1989;264(30):18083–90. 2808365

