# Supplementary material for: Cs1, a Clonorchis sinensis-derived serodiagnostic antigen containing tandem repeats and a signal peptide
Source: PLoS Negl Trop Dis. 2018 Aug 2;12(8):e0006683. doi: 10.1371/journal.pntd.0006683 (PMC6091968; doi:10.1371/journal.pntd.0006683)
Supplement: S1 Fig — (PDF) [file pntd.0006683.s001.pdf]

IPD-2009-14

中国疾病预防控制中心寄生虫病预防控制所
实验动物福利伦理审查申请表

National Institute of Parasite Diseases Chinese Center for Disease Control and Prevention

The Application Form for Animal Experimental Welfare & Ethical Inspection

一、基本信息 (Basic Information)

实验项目名称 Name of Research Project	基于功能基因组的重要寄生虫病防治基础科研支撑平台(2009ZX10004-302) Parastitic Diseases basic research support platform based on the importance of functional genomics technologies		
项目负责人姓名 Name of Applicant	胡薇 Hu Wei	职称/职务 Professional title	研究员 Professor
电话/传真 Telephone/FAX	021－64735439	电子邮件 E-mail	huweixf@gmail.com
部门名称 Name of Department	重点实验室 Key Laboratory of Parasite and Vector		
项目的意义及必要性 Importance of Research Project	本研究进行血吸虫、华支睾吸虫等寄生虫动物感染模型的建立，从而加速重要寄生虫病防治技术研究进程。 The establishment of animal models infected with *Schistosoma japonicum*, *Clonorchis sinensis*, etc. In order to promote the research of prevention and control technologies for important parasitic diseases.		
实验周期 Experimental periods	自：2009 年 1 月 1 日 至：2010 年 12 月 31 日		

二、直接进行动物实验的人员资料 (Information of Personnel responsible for animal care & use):

姓名 Name	性别 Gender	职称/职务 Professional title	岗位证书编号 No. of Certification	实验动物经验 Experience of Animal Experiment
徐斌 Xu Bin	女 F	副研究员 Associate professor		■有 Experienced<br>☐需要带教 Under the supervision of other Experienced personnel
莫筱瑾 Mo Xiaojin	女 F	助理研究员 Research associate		■有 Experienced<br>☐需要带教 Under the supervision of other Experienced personnel
周岩 Zhou Yan	女 F	研究实习员 Research assistant		■有 Experienced<br>☐需要带教 Under the supervision of other Experienced personnel
鞠川 Ju Chuan	男 M	博士研究生 Ph.D. candidate		■有 Experienced<br>☐需要带教 Under the supervision of other Experienced personnel

由 扫描全能王 扫描创建

三、 **实验动物信息 (Laboratory Animals Information):**

动物品种/品系 Animal Species	年龄/体重 Age/Weight	数量（Quantity）		供应单位 Supplier
		♀	♂	
1. BALB/c mice	18-22	200	0	SLAC, Shanghai
2. C57 mice	18-22	400	0	SLAC, Shanghai
3. New Zealand white rabbits	2.2-2.5kg	0	100	SLAC, Shanghai

四、 **动物饲养(Animal breeding):**

■由动物中心专人负责（Charging by special person in the animal center）；

☐由实验室人员负责，负责人姓名（Charging by lab staff, name of principle）：________________

五、 **动物实验的主要内容及病原（The Main Content and pathogen of the Animal Experiment）：**

接种血吸虫、华支睾吸虫等寄生虫或抗原

The mice or rabbits will be experimentally infected with *Schistosoma japonicum*, *Clonorchis sinensis*, etc or immunized with antigens.

六、 **简述下列实验步骤(Describe the following experiment step in brief):**

(1) 使用麻醉药品名称及麻醉方式（Name and methods of Anaesthesia）

乙醚 diethyl ether 或者 or CO_2

注：使用超过一种实验动物时，须分别填报每种动物所使用的药品剂量（In the occasion that more than one sort of animal is needed, Please fill out the leechdom dosage that each sort animal is used.）。

无须麻醉原因（The reason of no Anaesthesia）：________________________

(2) 若动物需长时间保定超过四小时，请说明所用器械与方法（If animal should be implemented restraint for more than 4 hours, Please describe the Methods of restraint）：

保定器 noose

七、 **请说明实验结束后动物的处置方式(Please explain the disposal methods of animal after experiment):**

(1) 实验后动物疼痛处理及照顾办法(The tendance methods for animal after experiment):

实验中不涉及此步骤 No this step

(2) 安乐死的方法(Methods of euthanasia):

麻醉后颈椎脱臼处死法 Cervical dislocation after naesthesia

(3) 实验动物尸体处理办法(Disposal of carcase):

■委托动物中心处理（Consign it to animal center） ☐其他（Others）：________________

本人保证以上所填数据完全属实（I assure that the above date is true.）

项目负责人签字(Signature of Applicant): [signature]

填报日期(Date) 2009 年 (YYYY) 2 月(MM) 16 日 (DD)

由 扫描全能王 扫描创建

审查结果（Result of Inspection）

☑通过 Approve

☐修正通过 Approve in terms of Modification

修正意见：<br>Opinions	

☐不通过 Disapprove

建议事项：<br>suggestions	

中国疾病预防控制中心寄生虫病预防控制所实验动物福利伦理审查委员会

Laboratory Animal Welfare & Ethics Committee (LAWEC)，National Institute of Parasitic Diseases of China CDC

主席（或授权人）签章（Chairman's Signature）：

日期(Date)：2009年（YYYY）2月(MM)23日（DD）

由 扫描全能王 扫描创建
