# Supplementary material for: Cs1, a Clonorchis sinensis-derived serodiagnostic antigen containing tandem repeats and a signal peptide
Source: PLoS Negl Trop Dis. 2018 Aug 2;12(8):e0006683. doi: 10.1371/journal.pntd.0006683 (PMC6091968; doi:10.1371/journal.pntd.0006683)
Supplement: S2 Fig — (PDF) [file pntd.0006683.s002.pdf]

中国疾病预防控制中心寄生虫病预防控制所

中国疾病预防控制中心寄生虫病预防控制所

（世界卫生组织疟疾、血吸虫病、丝虫病合作中心）

伦 理 审 查 委 员 会

NATIONAL INSTITUTE OF PARASITIC DISEASES (NIPD), CHINESE CENTER FOR DISEASE CONTROL AND PREVENTION (WHO COLLABORATING CENTRE FOR MALARIA, SCHISTOSOMIASIS AND FILARIASIS)

ETHICAL REVIEW COMMITTEE

Approval Notice

PRINCIPAL INVESTIGATOR OF PROJECT: Zhou Xiaonong

TITLE OF PROJECT: National S & T Major Program (Grant No. 2012ZX10004-220)

INSTITUTE: National Institute of Parasitic Diseases, China CDC

The Ethical Review Committee of National Institute of Parasitic Diseases, Chinese Center for Disease Control and Prevention has reviewed the proposal of "National S & T Major Program (Grant No. 2012ZX10004-220)". This project only deals with collecting elementary information about social, environmental, household and individual characteristics, collecting biological materials including feces and blood samples of human beings. It is recognized that all those methods for survey and samples collecting will be well accepted by the local people, the right and the welfare of the subject are adequately protected and the potential risks are outweighed by potential benefits.

SIGNATURE: [signature]

Tang linhua, MD, Professor

Chair, Ethical Review Committee, NIPD, China CDC

August 26, 2012

由 扫描全能王 扫描创建

中国疾病预防控制中心寄生虫病预防控制所

中国疾病预防控制中心寄生虫病预防控制所
（世界卫生组织疟疾、血吸虫病、丝虫病合作中心）
伦 理 审 查 委 员 会
THE NATIONAL INSTITUTE OF PARASITIC DISEASES (NIPD) ,CHINESE CENTER FOR DISEASE CONTROL AND PREVENTION（WHO COLLABORATING CENTRE FOR MALARIA, SCHISTOSOMIASIS AND FILARIASIS）ETHICAL REVIEW COMMITTEE

批 准 通 知 书

项目总负责人：周晓农
项目名称：国家科技重大专项(Grant No. 2012ZX10004-220)
承担单位：中国疾病预防控制中心寄生虫病预防控制所

20120826

中国疾病预防控制中心寄生虫病预防控制所伦理审查委员会已对“国家科技重大专项(Grant No. 2012ZX10004-220)”项目进行了评审（书面函审）。认为该项研究涉及居民血液、粪便采集中，所涉及的人群的利益已得到了充分的保护，受试者可能获得的利益超过可能的风险。

主任委员（签名）[signature]

中国疾病预防控制中心寄生虫病预防控制所

（代章）

2012 年 8 月 26日

地址：上海市瑞金二路207号
邮编：200025
电话 总机：21-64377008
直线：21-64376308
传真：86-21-64332670

由 扫描全能王 扫描创建
