# Supplementary figures and images for: Cs1, a Clonorchis sinensis-derived serodiagnostic antigen containing tandem repeats and a signal peptide
Source: PLoS Negl Trop Dis. 2018 Aug 2;12(8):e0006683. doi: 10.1371/journal.pntd.0006683 (PMC6091968; doi:10.1371/journal.pntd.0006683)

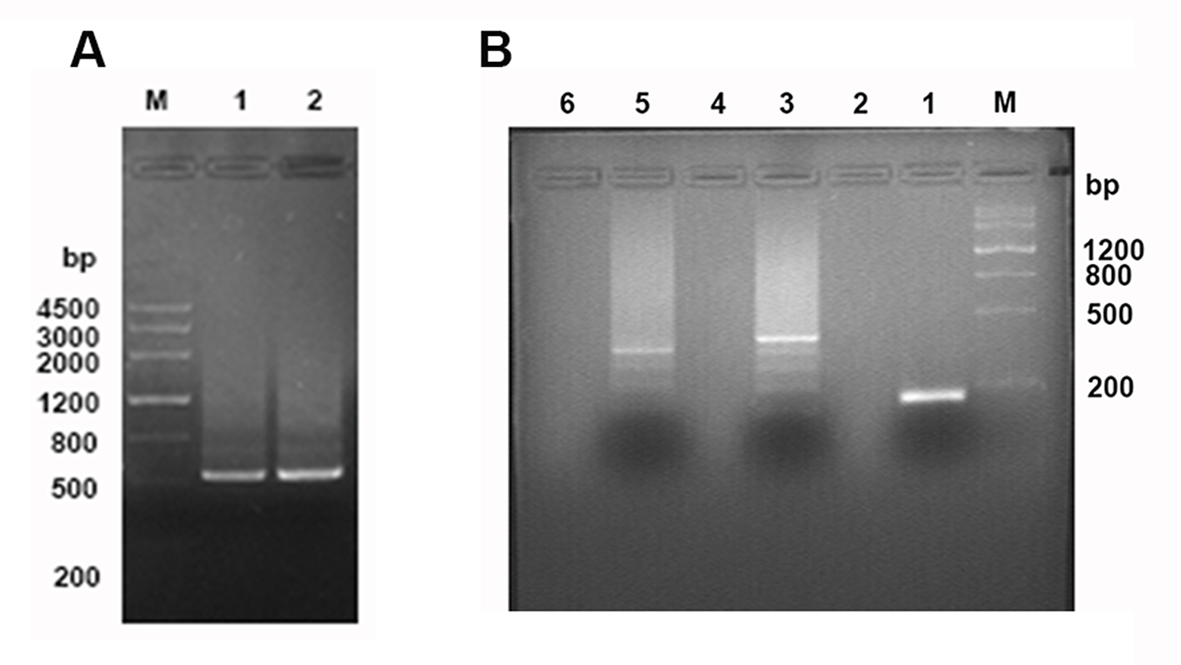

Supplement: S3 Fig — (A) Cs1 (without signal peptide). (B) The complete TR region, N-terminus (without signal peptide), and C-terminus plus TR region of Cs1. M, molecular weight marker. (TIF) [file pntd.0006683.s003.tif]
