# Supplementary material for: Cs1, a Clonorchis sinensis-derived serodiagnostic antigen containing tandem repeats and a signal peptide
Source: PLoS Negl Trop Dis. 2018 Aug 2;12(8):e0006683. doi: 10.1371/journal.pntd.0006683 (PMC6091968; doi:10.1371/journal.pntd.0006683)
Supplement: S1 Table — (DOCX) [file pntd.0006683.s004.docx]

| **Screened Genes** | **No. Positive Clones** | **Typical Clone(s)** | **Novel or Known** | **TRs** | **SP** |
| --- | --- | --- | --- | --- | --- |
| Glycine rich antigen 2a | 30 | Cs4 | Reported in our previous studies | Yes | Yes |
| Glycine rich antigen 1 | 7 | Cs7 | Known | Yes | Yes |
| PPMP antigen | 3 | Cs2, Cs3 | Reported in our previous studies | Yes | Yes |
| Cysteine proteinase | 2 | Cs12 | Known | No | Yes |
| Cs22 | 1 | Cs22 | Reported in our previous studies | Yes | Yes |
| Cs1 | 1 | Cs1 | Novel | Yes | Yes |
| TRs. tandem repeats; SP, signal peptide | | | | | |
